# Toxic Intraocular Syndrome

**DOI:** 10.18502/jovr.v17i1.10184

**Published:** 2022-01-21

**Authors:** Alfredo Amigó, Paula Martinez-Sorribes

**Affiliations:** Instituto Oftalmológico Amigó, Santa Cruz de Tenerife, Spain

Over the last three decades, several noninfectious postoperative inflammatory syndromes have been described: toxic anterior segment syndrome (TASS),^[1]^ fibrin syndrome, sterile endophthalmitis, and toxic posterior segment syndrome (TPSS).^[2,3]^ All have a common toxic origin but none describe all the clinical forms in which toxicity can occur and there are cases of toxicity that do not fit into any of these syndromes. This descriptive limitation can lead to confusion and delay a diagnosis that is essential in the prevention of new cases of ocular toxicity.

The toxic intraocular syndrome (TIOS) that we describe encompasses all forms of toxicity described, referring to any postoperative intraocular inflammation, due to a noninfectious substance, which can occur after any type of intraocular surgery, resulting in toxic damage to any segment of the eye.

Postoperative intraocular toxicity is an underdiagnosed and underreported entity, with a reported frequency ranging from 0.2%^[4]^ to 2.0%,^[6]^ higher than that of infectious endophthalmitis.

The most frequent clinical manifestation of TIOS consists of an early and painless postoperative decrease in vision. The operated segment is usually the most affected, however, there are mixed forms with involvement of both segments,^[6,7]^ as well as paradoxical forms with inflammatory involvement of the segment opposite to the intervened segment.^[8,9,10]^ When the anterior segment (AS) is affected, the predominant sign is limbus-to-limbus corneal edema to a variable degree, which may be accompanied by iritis, fibrin bands, hypopyon, and/or pupillary paresis. TASS is a form of TIOS in which the signs and surgery are limited to the AS. When the posterior segment (PS) is involved, the signs are more polymorphic, the vitreous may be clear or with the signs of vitritis, the retina may be intact or be associated with hemorrhages, vasculitis, pigment epithelium lesions, and macular edema. Occasionally, early papillary pallor with optic atrophy may appear. TPSS is a form of TIOS in which after PS surgery the findings are limited to this segment.

Every substance involved in the surgical process has been reported as a cause of operative toxicity. Those related to the cleaning of microcannulated instruments, enzymatic soaps, endotoxins, intraocular dissolutions, denatured viscoelastic, perfluorocarbons, silicone, are among many others.

The most important differential diagnosis is infectious endophthalmitis from which TIOS tends to be differentiated by presenting as a cold and early postoperative inflammation, with decreased vision and no other symptoms. The absence of initial pain, hyperemia, lid edema, or swelling may help to differentiate TIOS from infectious endophthalmitis. Cultures, if taken, will always be negative. The prognosis of TIOS will depend on the type of toxin, its concentration, and time of exposure, ranging from complete spontaneous resolution to irreversible loss of vision or loss of the eyeball.

There is no specific treatment for TIOS with prevention being the best current treatment. Suspecting toxicity, as a possible origin of any postoperative inflammation, is essential in the prevention of new cases given the frequent presentation of TIOS in the form of an outbreak in the following surgical sessions.

##  Financial Support and Sponsorship

Nil.

##  Conflicts of Interest

There are no conflicts of interest.
